# Communication, advice exchange and job satisfaction of nursing staff: a social network analyses of 35 long-term care units

**DOI:** 10.1186/1472-6963-11-140

**Published:** 2011-06-01

**Authors:** Adriana PA van Beek, Cordula Wagner, Peter PM Spreeuwenberg, Dinnus HM Frijters, Miel W Ribbe, Peter P Groenewegen

**Affiliations:** 1 NIVEL: Netherlands Institute for Health Services Research, PO BOX 1568, 3500 BN Utrecht, The Netherlands; 2 EMGO+, Department of Public and Occupational Health, VU University Medical Center, Van der Boechorststraat 7, 1081 BT Amsterdam, The Netherlands; 3 EMGO+, Department of Nursing Home Medicine, VU University Medical Center, Van der Boechorststraat 7, 1081 BT Amsterdam, The Netherlands; 4 Utrecht University, Department of Sociology and Department of Human Geography, PO BOX 80.115, 3508 TC Utrecht, The Netherlands

## Abstract

**Background:**

The behaviour of individuals is affected by the social networks in which they are embedded. Networks are also important for the diffusion of information and the influence of employees in organisations. Yet, at the moment little is known about the social networks of nursing staff in healthcare settings. This is the first study that investigates informal communication and advice networks of nursing staff in long-term care. We examine the structure of the networks, how they are related to the size of units and characteristics of nursing staff, and their relationship with job satisfaction.

**Methods:**

We collected social network data of 380 nursing staff of 35 units in group projects and psychogeriatric units in nursing homes and residential homes in the Netherlands. Communication and advice networks were analyzed in a social network application (UCINET), focusing on the number of contacts (density) between nursing staff on the units. We then studied the correlation between the density of networks, size of the units and characteristics of nursing staff. We used multilevel analyses to investigate the relationship between social networks and job satisfaction of nursing staff, taking characteristics of units and nursing staff into account.

**Results:**

Both communication and advice networks were negatively related to the number of residents and the number of nursing staff of the units. Communication and advice networks were more dense when more staff worked part-time. Furthermore, density of communication networks was positively related to the age of nursing staff of the units. Multilevel analyses showed that job satisfaction differed significantly between individual staff members and units and was influenced by the number of nursing staff of the units. However, this relationship disappeared when density of communication networks was added to the model.

**Conclusions:**

Overall, communication and advice networks of nursing staff in long-term care are relatively dense. This fits with the high level of cooperation that is needed to provide good care to residents. Social networks are more dense in small units and are also shaped by characteristics of staff members. The results furthermore show that communication networks are important for staff's job satisfaction.

## Background

Studies have found that cohesive groups of nursing staff are related to higher work satisfaction and quality of care [1], and lower anticipated turnover [2]. Yet, at the moment little is known about the social structure of nursing staff relations. This is the first study to investigate informal social networks of nursing staff in long-term care, based on social network analyses.

Social networks in organisations in which individuals are embedded affect behaviour [3], and are important for the diffusion of information and the influence of employees [4]. Studies have shown that social networks are beneficial for career advancement [5,6], job performance [7], and diminishment of conflict [8]. Until now, studies on social networks have mainly concentrated on the business sector or small groups of professionals. Not much is known about the role of social networks in healthcare settings although Coleman as early as in 1957 found that social networks of medical doctors were conducive to their prescription of new drugs [9]. West et al. [4] described in their study the social networks of clinical directors of medicine and directors of nursing in hospitals. They found that the networks of both types of directors differ. Networks of directors of nursing were more hierarchical and highly central. Directors of medicine often worked in tightly knitted networks or cliques. Kravitz et al. [10] identified opinion leaders on caesarean delivery in obstetric care by studying the advice networks of obstetricians, family physicians and nurse midwives. Heiligers et al. [11] studied the impact of part-time work on the networks of doctors in hospitals. They found that working part-time did not influence the personal social networks of doctors. Nonetheless, they found that there were less communication ties in mixed teams of part-time and full-time working doctors compared to teams of full-time doctors only. Creswick et al. [12] looked at the social networks of staff working in Emergency Departments, including doctors, nurses and allied health professionals. Their results showed that individuals were most closely connected to colleagues of the same profession. Cott [13] studied the social networks of three multidisciplinary teams of healthcare workers, including nurses, in a geriatric care facility. The author found that multidisciplinary teamwork increased participation in decision-making only for the higher status professionals. The hierarchal structure of the teams did not change.

Krackhardt & Hanson [14] distinguish three types of informal social networks in organizations: communication, advice and trust networks. Communication networks consist of employees who talk about work-related matters on a regular basis. The advice network is formed by the prominent players in an organization, the employees on which others depend to solve problems and exchange information. The trust network shows which participants share delicate information and back one another in a crisis. In this article, we study communication and advice networks of nursing staff in long-term care dementia settings in the Netherlands.

The group of elderly persons with dementia who need intensive long-term care will increase significantly in years to come. In 2010, there were approximately 35.6 million people with dementia worldwide. This number will increase to 115.4 million in 2050 [15]. In the Netherlands, there are around 235.000 persons with dementia of which 35% live in a long-term care facility [16]. The Dutch work population is projected to decline. It is therefore particularly important to understand how care for persons with dementia can be optimized.

Facility based care for persons with dementia in the Netherlands is provided in nursing homes and residential homes in special (psychogeriatric) units [17]. Residents who live on these units often share their bedroom and receive multidisciplinary care. The doors on the units are closed for residents, so that they cannot leave the unit on their own accord. In addition, in residential homes care for persons with dementia is provided in day-time psychogeriatric group projects which aim to delay or prevent admission of residents to a nursing home. Residents who attend these group projects live in their own apartments in the facility, but spend most of their day in a small group-setting of approximately 10 to 12 residents [18] most often in a living room designated to this purpose. Group projects provide multidisciplinary care to residents and aim to provide a daytime routine and activities with other residents in a sheltered setting [19].

The majority of nursing staff in Dutch long-term care consists of Certified Nurse Assistants (CNAs), who generally have three years of basic nursing training, and perform most care tasks [20]. Furthermore care is provided by care assistants, trainees and some fully qualified nurses. In group projects recreational therapists are also often employed.

First, we look at the structure of communication and advice networks. Second, we study how these networks are related to the size of the care units and characteristics of nursing staff. McPherson & Smith-Lovin [21] have found that social network patterns are influenced by the relative size of groups. Characteristics of nursing staff may also influence the structure of social networks. For example, nursing staff in Dutch long-term care facilities often work part-time, which limits the possibilities to meet colleagues. Third, we explore if social networks are related to the job satisfaction of nursing staff.

The following research questions are addressed:

1. What is the structure of communication and advice networks of nursing staff in long-term care?

2. Are social networks of nursing staff related to the size of units and characteristics of staff members?

3. Are social networks of nursing staff related to job satisfaction?

## Methods

Data were gathered on 37 units for residents with dementia in nursing homes and residential homes in the Netherlands in psychogeriatric units and group projects during October 2002 - June 2003 (on one unit/project per facility). In total, 26 psychogeriatric units participated in the research of which 16 units in nursing homes and 10 units in residential homes. In residential homes 11 group projects took part. Facilities were asked to participate in the study on a voluntary basis.

### Size of units and characteristics of nursing staff

Data on the number of residents and nursing staff on the units was collected in an interview with the unit-supervisor at the beginning of data collection on each unit. Characteristics of nursing staff were measured with a questionnaire for all nursing staff on the units. We asked staff their age, gender, and the number of years they worked on the unit. In addition, we asked if they worked full-time or part-time and if they had a permanent position.

### Measurement of social networks

We individualized the questionnaires for each unit by presenting the names of all staff. To measure communication networks we asked the following question: *'Please report for each colleague how often you speak to him or her about your work or things that happen at work' *[14]. The frequency of contacts had a range from a few times a day (5) to less than two times a month/never (0). Responses of individual members of nursing staff were symmetrised [22,23], coding a tie between two members of nursing staff (dyads) when at least one indicated a relation with the other. The answers for communication networks were then dichotomized [22,24] into at least once a week and rare (twice a month or less).

Advice networks were measured with the question: *'Sometimes we all need advice on how to best do our job. Who comes to you for advice on this unit and how often?' *For this question, the frequency of contacts also ranged between a few times a day (5) to less than two times a month/never (0). For the advice network we also recorded the direction of ties. Therefore, answers for this network were not symmetrised. We expected advice exchange to be less frequent than communication. For this reason answers for advice networks were dichotomized into frequent (at least two times a month) and rare (less often or never).

### Job satisfaction

Job satisfaction of nursing staff was measured with the Maastricht Work Satisfaction Scale for Healthcare (MAS-GZ) [25]. The MAS-GZ consists of 21 items which have to be scored on a five-point scale ranging from very dissatisfied (1) to very satisfied (5). Items address the satisfaction with the unit supervisor, promotion possibilities, quality of care, contacts with colleagues and residents, and clarity of tasks. For this study, one item ('the extent to which you can get ahead in the facility') was removed as it is not directly related to working on the unit. Internal consistency (Cronbach's alpha) of the 20 remaining items in our sample was 0.88.

### Data analysis

Answers on the social network questions were analyzed using the UCINET software package [22], which models the relationships of subjects in a certain group. With social network analysis several aspects of networks can be measured. For instance, Völker and Flap [26] name four dimensions of social networks that are important for organizational performance. These dimensions are the number of ties, the quality, the hierarchy, and the density of the network. In this article, we focused on the density (or number of contacts) both for communication and advice networks. Density is a measure of the general level of cohesion of the network on the unit. It describes the extent to which actors are tied to each other [4]. Density has a value between 0 and 1. A density of 1 represents a saturated network: all members of nursing staff interact with each other. With a density of 0 none of the nursing staff interacts [22]. Aggregated data for the units were analyzed in SPSS 18.0. Differences between types of care unit were analyzed using Kruskal-Wallis Tests. The relationship between social networks, size of units and characteristics of nursing staff were examined through Spearman's rho correlations. Last, we investigated the relationship between social networks and job satisfaction using multilevel analyses [27,28] in the statistical package MLwiN, with job satisfaction as the main dependent variable. We analyzed a random intercept model with two levels: units (level 2) and nursing staff on these units (level 1). After the empty model, we first entered characteristics of nursing staff into the model (model 1). Second, we entered type of care-setting and number of nursing staff one after another into the model (model 2). Third, density of communication and advice networks were entered separately in to the analyses (model 3). As our study is mainly explorative in nature, we decided to use a significance level of p < 0.10.

## Results

### Size of units and characteristics of nursing staff

The number of residents of the units varied from 8 to 34, with an average of 21 residents. The group projects provided care to an average of 12 residents, compared to 22 in psychogeriatric units in residential homes and 27 residents in units in nursing homes. The number of nursing staff in the units varied from 4 to 39, with an average of 23. Group projects in residential homes had on average 11 staff members, compared to 26 staff members in psychogeriatric units in residential homes and 30 in psychogeriatric units in nursing homes.

A total of 474 staff members completed the questionnaire. This was 55% of all 861 nursing staff of the units. Almost half of all respondents were CNAs (46%). Other respondents were care-assistants, trainees, nurses and recreational therapists. The majority of the responding staff members were women (95%), with an average age of 38 years (sd = 10.6). Nursing staff mostly worked part-time (77%) and the vast majority held a permanent position (89%). Nursing staff worked on average between 4 and 5 years in the units, varying between one month and 28 years (see Table 1). There were no significant differences between nursing staff in the different care-settings for the percentage of women, their average age, the percentage of nursing staff that worked part-time and the percentage of nursing staff with a permanent position. There were differences, however, in the average number of years nursing staff worked in the units and group projects. Half of the nursing staff in residential homes, both of psychogeriatric units and group projects, had been working in the unit for one to three years compared to 28% of nursing staff in nursing homes. In nursing homes more nursing staff worked on the unit for ten years or longer (not in Table).

**Table 1:** Characteristics of nursing staff in the care settings and in total (N = 474).

	Psychogeriatric units in nursing homes (N = 246)	Psychogeriatric units in residential homes (N = 141)	Group projects in residential homes (N = 87)	Total (N = 474)
Age (mean and sd*)	37.5 (10.0)	38.9 (11.0)	39.3 (11.8)	38. 2 (10.6)
Women (%)	94.3	96.5	96.5	95.3
Part-time (%)	74.2	82.7	81.4	78.0
Permanent position (%)	87.3	92.9	88.5	89.2
Years on unit (mean and sd)	5.4 (5.5)	3.8 (4.6)	3.5 (3.7)	4.6 (5.0)

* = standard deviation

Analyses showed that the 37 units that participated in the study did not differ from all nursing homes and residential homes in the Netherlands in the age and qualifications of nursing staff [29].

### Response rates on social network measures

The question on communication was answered by 380 staff members. Due to a low response rate (less than 15%) two units were excluded from further analysis. The response percentage on the remaining 35 units with 801 staff members varied between 18% and 100% per unit with an average response rate of 53%. On psychogeriatric units in nursing homes and residential homes the average response rate was 45%. In group projects the average response rate was 71% (see Table 2).

**Table 2:** Response rates and density of social networks of nursing staff of the units (N = 35).

Networks	Psychogeriatric units in nursing homes (N = 14)	Psychogeriatric units in residential homes (N = 10)	Group projects in residential homes (N = 11)	p
**Response in %**				
Communication networks (mean and sd)	44.7 (14.0)	45.3 (14.6)	71.0 (31.0)	0.149
Advice networks (mean and sd)	41.3 (13.7)	41.8 (12.2)	67.6 (29.5)	0.079
**Density**				
Communication networks (mean and sd)	0.44 (0.1)	0.43 (0.2)	0.69 (0.3)	0.014
Advice networks (mean and sd)	0.20 (0.1)	0.22 (0.1)	0.38 (0.2)	0.034

The question on advice networks was answered by 347 staff members of the 35 units. The response rate between the units varied from 18% to 100%, with an average rate of 50%. The average response rate for group projects was 68%. For psychogeriatric units in nursing homes and residential homes response rates were respectively 41% and 42%.

Response rates on the communication networks and advice network questions were highly correlated (0.97, p < 0.001). The response rate was negatively related to the size of the units. On units with more residents, response rates on communication and advice networks were lower (correlation between the number of residents and the response rate on communication networks was -0.43 and advice networks -0.47, p < 0.01). Similar results were found for units with more nursing staff (correlation between the number of nursing staff and the response rate on communication networks was -0.61, for advice networks -0.61, p < 0.001).

### Density of communication and advice networks

**Figure 1:**
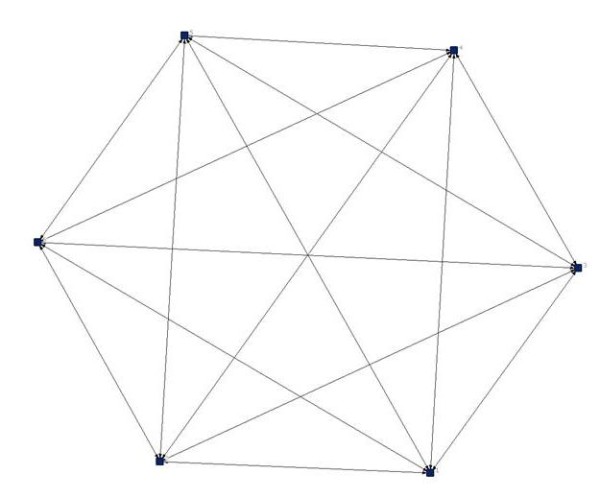
**Example of a communication network in a group project of a residential home with 6 staff members (response rate 100%), density is 1.0**.

**Figure 2:**
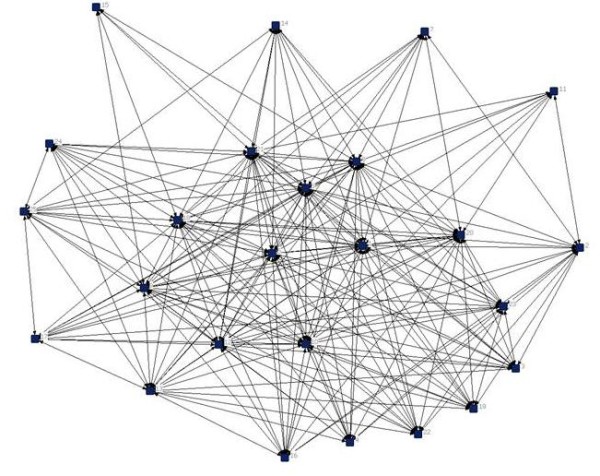
**Example of a communication network in a psychogeriatric unit of a residential home with 25 staff members (response rate 60%), density is 0.67**.

For communication networks we looked at the weekly contacts among staff members. Figures 1 and 2 provide examples of communication networks of nursing staff in a group project and in a psychogeriatric unit, as illustrated with NetDraw [21]. Example A shows a completely saturated network of a group project; all staff members communicate with each other. Example B shows a network of a psychogeriatric unit, with a density of 0.67. Example B clearly shows that it is more difficult to communicate with all other members of staff of larger units in comparison with smaller units. The average density of the communication networks for all units was 0.52, varying between 0.22 and 1.0. Overall, communication networks in group projects had a higher density than networks in psychogeriatric units (Table 2).

**Figure 3:**
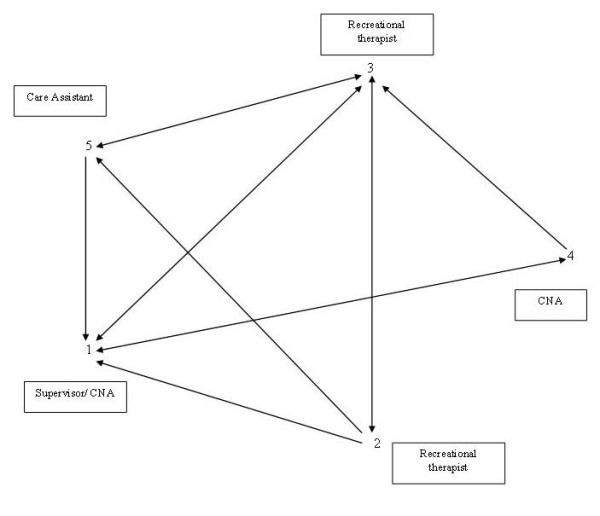
**Example of an advice network in a group project with 5 staff members (response rate 100%), density is 0.60**.

For advice networks, we studied monthly contacts between staff members. Figure 3 gives an example of an advice network of a group project with 5 staff members. Although all nursing staff communicated with each other, the density of the advice network was less strong (0.60). Staff member 4 only exchanged advice with staff member 1 and 3. The other staff members 2 and 5 had no ties with 4, although they exchanged advice with staff members 3 and 1. Figure 2 also shows that all staff ask advice from the supervisor of the group project. An equal position is taken by staff-member 3, one of the recreational therapists.

Overall, advice networks were smaller than communication networks and had an average density of 0.26, varying between 0.09 and 0.83 between the units. Identical to communication networks, advice networks in group projects had a higher density than networks in pyschogeriatric units. This difference was significant (Table 2).

### Social networks, size of units and characteristics of nursing staff

Table 3 gives an overview of the correlations between the social networks, size of the units, characteristics of staff members, and work-related outcomes for the 35 units. Both communication and advice networks were negatively related to the number of residents and the number of nursing staff of the units (p < 0.01). Consequently, on units with more residents and nursing staff, staff communicated less with each other and less often asked each other for advice. Communication and advice networks were more dense when more staff worked part-time (p < 0.10). Density of communication networks was also positively related to the age of nursing staff of the units (p < 0.10).

**Table 3:** Correlations between social networks and characteristics of units/nursing staff (N = 35).

	Density of advice network	Number of residents	Number of staff members	Average age nursing staff	Average time on the unit (in months)	Percentage of staff with a permanent position	Percentage of staff working part-time
Density of communication network	0.854***	-0.445***	-0.698***	0.306*	-0.078	0.111	0.314*
Density of advice network		-0.522***	-0.687***	0.259	-0.085	0.164	0.295*
Number of residents			0.712***	-0.374**	0.390**	-0.175	-0.337**
Number of nursing staff				-0.385**	0.237	-0.270	-0.330*
Average age nursing staff					-0.095	0.013	0.500***
Average time on the unit (in months)						0.233	-0.100
Percentage of staff with a permanent position							0.084

***: correlation is significant at the 0.01 level; **: correlation is significant at the 0.05 level; *: correlation is significant at the 0.10 level.

Units with dense communications networks also had dense advice networks (p < 0.01). The size of units was also related to characteristics of staff. In units with more residents and staff members, nursing staff were younger (p < 0.05), and less often worked part-time (p < 0.10).

### The relationship between social networks of nursing staff and job satisfaction

Overall job satisfaction of nursing staff of the 35 units was 3.76 (sd = 0.19) ranging from 3.12 to 4.33 for the units. Multilevel analyses showed that job satisfaction differed significantly between individual staff members and units (see variance components for the empty model in Table 4). We first entered characteristics of nursing staff into the model on the level of individual staff members (model 1). None of these characteristics were found to be related to job satisfaction in our sample. By taking characteristics of nursing staff into account, 15% of the variance could be ascribed to differences on the level of the units (as presented by the intra-class correlation in model 2, Table 4). Next, we entered characteristics of the units into the model on unit-level. Type of care setting was not significantly related to job satisfaction of nursing staff (not in Table). Yet, a significant relationship was found between job satisfaction and the number of nursing staff on the units (p < 0.05). On units with less staff members, nursing staff were more satisfied with their job (see model 2, Table 4). Finally, density of communication and advice networks were entered into the model on the level of the units (model 3). Only communication networks were positively related to job-satisfaction (p < 0.10); on units where communication networks were more dense, nursing staff were more satisfied with their job. By adding communication networks into the model, the relationship between the number of nursing staff of the unit and job satisfaction ceased to exist. No effect was found for the density of advice networks of nursing staff (not in Table). By adding density of communication networks into the model, the variance that could be ascribed to differences on the level of the units decreased to 12% (as presented by the intra-class correlation in model 3, Table 4).

**Table 4:** Results of the multilevel analyses for job satisfaction of nursing staff (N = 410) on the units (N = 35).

Job satisfaction	Empty model		Model 1		Model 2		Model 3	
	B	(SE)	B	(SE)	B	(SE)	B	(SE)
Intercept	**3.758**	**(0.026)**	**3.757**	**(0.027)**	**3.905**	**(0.068)**	**3.604**	**(0.174)**
**Characteristics of nursing staff**								
Age			0.001	(0.002)	0.000	(0.002)	0.000	(0.002)
Gender			-0.068	(0.081)	-0.063	(0.081)	-0.063	(0.081)
Years on unit			-0.003	(0.003)	-0.002	(0.003)	-0.002	(0.003)
Permanent position			-0.057	(0.051)	-0.062	(0.051)	-0.061	(0.051)
Working fulltime			0.041	(0.039)	0.044	(0.039)	0.044	(0.039)
**Number of nursing staff**					-0.006**	**(0.003)**	-0.001	(0.004)
**Communication networks**							0.352*	**(0.188)**
**Variance components**								
Units	**0.015**	**(0.006)**	**0.016**	**(0.006)**	**0.013**	**(0.005)**	**0.012**	**(0.005)**
Nursing staff	**0.090**	**(0.007)**	**0.088**	**(0.006)**	**0.088**	**(0.006)**	**0.088**	**(0.006)**
ICC units	14%		15%		13%		12%	

** significant association at p < 0.05, * significant association at p < 0.10.

## Discussion

In this study, we first looked at the structure of communication and advice networks of nursing staff in long-term care. Overall, social networks were relatively dense. Nursing staff of group projects communicated more often with each other than nursing staff of psychogeriatric units. Nursing staff of group projects also exchanged more advice with each other.

Second, we studied if social networks were related to the size of units and characteristics of staff members. As could be expected, differences in social networks were mainly due to the number of nursing staff of the units. Group projects provide care to a smaller group of residents than psychogeriatric units and, hence, have less nursing staff. As a consequence, it is probably easier to communicate with all staff members in this setting. This relationship between the size of a group and the structure of its social network is also found in other studies [21]. Furthermore, we found that communication and advice networks were denser when more staff members worked part-time. This finding is in contrast to the study of Heiligers et al. [11]. This difference may be due to the fact that the majority of the nursing staff in our sample worked part-time whilst most doctors in the study of Heiligers et al. worked full-time, indicating that differences in communication may not be ascribed to the differences in working full-time or part-time per se, but in belonging to the majority of part-time or full-time working employees in a group.

Third we investigated if communication and advice networks of nursing staff were related to job satisfaction. In our analyses job satisfaction was also related to the number of nursing staff of the units. However, this relationship disappeared when we took the density of communication networks into account. This finding corresponds with the results of Leppa [1] and shows the importance of studying social networks when investigating work-related outcomes of nursing staff.

The results also illustrate the importance of studying informal social networks instead of solely focusing on the formal networks of staff members. Figure 2 shows that both staff-member 1 and 3 are asked for advice by all other staff members, and therefore take a similar position in the advice network. However, staff member 1 is the unit-supervisor whereas staff member 3 is one of the recreational therapists. By mapping informal social networks it is possible to identify key-players within the organisation and, consequently, to disseminate information more effectively [14,30].

Our data should be interpreted with caution. An important problem in social network research is that data-collection is difficult and very time-consuming. It was not possible to obtain complete social network data of all 37 units, and because of paucity of data the social networks of 35 units were analyzed.

Communication networks in our sample had an average response rate of 53%; the average response on advice networks was 50%. This response rate is comparable with the response rate of 58% in the study of Kravitz et al. [10]. Kossinets [23] argues that non-response in social network surveys can be partially balanced out by reciprocal nominations of actors. If actor A does not fill in the network questionnaire but actors B and C of the same network describe their interactions with A, information about the social network of actor A is still available. In our analyses of the communication networks we symmetrised our data, coding a tie between members of nursing staff when at least one staff- member indicated a tie with this colleague. Through this, we also gained information on the communication networks of those who did not complete the questionnaire. In addition, it is found that social network centrality measures are relatively robust even when using imperfect data [31]. We found that response rates were lower in units with more nursing staff. We studied whether this relationship between the size of the unit and social networks could be due to differences in response, as we found that the response on the social network questions was lower in larger units. When we controlled for the average response on the social network questions, the relationship between the number of nursing staff and the density of communication and advice networks remained significant (respectively p < 0.01 and p < 0.05). However, the relationship between the number of residents and the density of social networks ceased to exist. Evidently it is easier to answer questions for a limited group of colleagues than for a larger group. Thereto, the response rates on the social network questions may also be seen as a dependent variable in this study as it appears to be structured by organizational aspects of long-term care. Nevertheless, missing data on the larger units in our sample form an important limitation for this study Further studies should focus on methods to obtain complete social network data of nursing staff. The relatively high level of non-response may also have consequences for our findings on job satisfaction, perhaps even leading to non-response bias [32]. Unfortunately, we have no information on the job satisfaction of non-respondents in our study. However, we find that our respondents did not differ from nursing staff in general in nursing homes and residential homes in the Netherlands, in terms of the percentage of females on the units, and their average age [33]. Furthermore, overall scores on job satisfaction are comparable with other findings in Dutch dementia care [34]. Concerning job satisfaction, we also identify another limitation. In this study we adjusted the MAS-GZ job satisfaction questionnaire for the specific setting by deleting one item. Although internal consistency of the remaining 20 items is good, this may have consequences for the questionnaires psychometric properties of the scale. For this reason, we studied how our results (with one item of the MAS-GZ missing) tied in with the findings of Merten et al. [34], in which data were collected of 236 staff members in Dutch dementia units. Average job satisfaction based on 21 items was 3,64 (sd = 0,43); for 20 items the average job satisfaction was 3,66 (sd = 0,43). It thus seems that the adjusted scale of job satisfaction results in a comparable score as when all 21 items were used.

Finally, we focused on the density of communication and advice networks as we were interested in social networks as a collective measure instead of the social networks of individual staff members (or dyads). Consequently, it is not possible to study if individual characteristics of staff members determine their social contacts. For instance, several studies have found that individuals are especially willing to form social connections with similar others [12,21,35]. This concept of homophily in social networks could also be applicable to nursing staff in long-term care settings. Furthermore, it would be interesting to investigate other aspects of networks, such as cliques that may be formed due to differences in work-shifts of nursing staff. In psychogeriatric units a selection of nursing staff work evening- and night-shifts to provide 24 hour care to residents. In group projects, nursing staff mostly only work day-shifts as activities are only provided during the day. This difference in shifts is likely to influence the formation of networks of staff. As yet, it is unclear if differences in social networks between the units in our sample can be ascribed to similarities between staff members or differences in work-shifts on the units.

Despite these limitations, this study is the first to study informal social networks of nursing staff in a large number of long-term care units. We focused on the number of connections (or density) between staff members as a base for information exchange in communication and advice networks. Burt [5], in his theory of structural holes, argues that networks with low density are more productive than networks with a high density. Networks with low density seem to be especially efficient in competitive work-settings. Tasks that depend on cooperation, on the other hand, profit from networks with a high density [36]. Our results support this finding. Overall density of the networks on the units is relatively high which is probably necessary when providing care for residents with dementia who, because of their problems in cognition, cannot clearly voice their needs and wishes.

## Conclusions

This article investigates communication and advice networks of nursing staff in long-term care for the first time, using a social network approach. The study demonstrates that communication between nursing staff is important for job satisfaction and that networks are not solely shaped by the formal positions of staff members. Further research is needed to investigate the nature of the relationship between informal social networks and other work-related outcomes of nursing staff and to investigate if social networks also influence care processes in long-term care.

## Competing interests

The authors declare that they have no competing interests.

## Authors' contributions

AB participated in the study design, collected data, performed the statistical analyses, interpreted the data and wrote this paper. PS helped with the interpretation of the data and the construction of the multilevel analyses. CW and PG participated in the design and planning of the study, the interpretation of data, and the critical revision of the manuscript. DF and MW critically revised the manuscript. All authors have given final approval of the submitted manuscript.

## Pre-publication history

The pre-publication history for this paper can be accessed here:

http://www.biomedcentral.com/1472-6963/11/140/prepub
